# Supplementary material for: A multiplexed, automated evolution pipeline enables scalable discovery and characterization of biosensors
Source: Nat Commun. 2021 Mar 4;12:1437. doi: 10.1038/s41467-021-21716-0 (PMC7933316; doi:10.1038/s41467-021-21716-0)
Supplement: Supplementary file 1 — Supplementary Information [file 41467_2021_21716_MOESM1_ESM.pdf]

Supplementary Table 1 | Selection parameters.

Selection	Prefixes	Suffix	Library	Order of Rounds	Ligation Method	Free Mg^{++}	Rounds	Hits (First Round)
**S1**	A, B	S	Loop1: N7<br>Loop2: N30	One -ligand selection round for cleavers, followed by alternating +/- ligand rounds.	Separate RT primer, splint, and ligation substrate	Low	57	Theophylline (R57)
**S2**	A, B, W	X	Loop1: N4-N8<br>Loop2: N30, N60<br>+<br>Loop1: N30, N60<br>Loop2: N4-N8	Four -ligand selection rounds for cleavers, followed by alternating +/- ligand rounds.	Single molecule RT primer/splint/ligation substrate	+4mM during RT setup after R46	64 (T2,T5), 78(T3), 100(T6)	(*S*)-reticuline (R36)
**S3**	W, Z	X	Loop1: N4-N8<br>Loop2: N30, N60<br>+<br>Loop1: N30, N60<br>Loop2: N4-N8	Two -ligand selection rounds for cleavers, followed by alternating +/- ligand rounds.	Single molecule RT primer/splint/ligation substrate	+4 mM during RT	114 (T1), 202(T2), 198(T3)	(*S*)-reticuline (R126), noscapine (R84), trans-zeatin (R126), aciclovir (R74)
**S4**	W, Z	X	Loop1: N4-N8<br>Loop2: N30, N60<br>+<br>Loop1: N30, N60<br>Loop2: N4-N8	Started with the pre-selection product (R2) from the prior selection (S3), followed by a repeating pattern of one +ligand round followed by two -ligand rounds.	Single molecule RT primer/splint/ligation substrate	Low	294(T1)	Gardiquimod (R102)

In above table, N*xx* refers to the number of degenerate nucleotides in each of the ribozyme loops; T*n* refers to ligand group *n*; S*n* refers to selection run *n*; R*n* refers to round *n* of a selection run. Low values for free Mg^{++} are <1mM.

Supplementary Table 2 | Sequences of oligonucleotides, prefixes, and suffixes.

ID #	Name	Description	Sequence
	W	Ribozyme prefix "W"	AAACAAACAAA
	Z	Ribozyme prefix "Z"	ACAAAACAAAAC
	A	Ribozyme prefix "A"	CTTTTCCGTATATCTCGCCAG
	X	Ribozyme suffix	AAAAAGAAAAATAAAAA
**BT88p**	T7	T7 RNA Polymerase promoter	AATTTAATACGACTCACTATA GGG
**BT575p**	Uncleaved Stop	RT Primer used for uncleaved selection; PCR reverse primer; qPCR reverse primer	TTTTTATTTTTCTTTTT GCTGT TTC GTCC
**BT1305p**	Asplint	RT primer/splint for A prefix addition	/5PHOS/G ACAGC CTGGCGAGATATACGGAAAAG AG GCUGT C ACCGGA TCCGGT CTGATGA GUCC TTTCTTTTT GCTGT TTC GTCC
**BT1316p**	Wsplint	RT primer/splint for W prefix addition	/5PHOS/G ACAGC TTTGTTTGTTTCCC AA GCUGT C ACCGGA TCCGGT CTGATGA GUCC TTTCTTTTT GCTGT TTC GTCC
**BT1508p**	Zsplint	RT primer/splint for Z prefix addition	/5PHOS/G ACAGC GTTTTGTTTTGT CCC AC GCUGT C ACCGGA TCCGGT CTGATGA GUCC TTTCTTTTT GCTGT TTC GTCC
**BT29p**	T7A Primer	PCR forward primer for A prefix; qPCR forward primer for A prefix	AATTTAATACGACTCACTATAGGGCTTTTCCGTATATCTCGCCAG
**BT1285p**	T7W Primer	PCR forward primer for W prefix	AATTTAATACGACTCACTATAGGG AAACAAACAAA GCTG
**BT1510p**	T7Z Primer	PCR forward primer for Z prefix	AATTTAATACGACTCACTATAGGG ACAAAACAAAAC GC
**BT1530**	W qPCR primer	qPCR forward primer for W prefix	CTATA GGG AAACAAACAAA GCTG
**BT1532**	Z qPCR primer	qPCR reverse primer for W prefix	CTATA GGG ACAAAACAAAAC GC
**BT1180p**	qPCR reference	qPCR reference	ATTGCGAGGGTGAGAATGAGG CTACACCCGACACGGTGGCGTGTTCTCTGAGGAATGCTCTTGGAGGTG GGTCTACTATCTCAAGCCGTA
**BT1178**	qPCR reffwd	qPCR reference forward primer	ATTGCGAGGGTGAGAATGAGG
**BT1179**	qPCR refrev	qPCR reference reverse primer	TACGGCTTGAGATAGTAGACC
**BT1440**	A_R6R30a_X-RC	NGS reference	CC TTTTCTTTTT GCTGT TTC GTCC TCGGCATAGC CAGAGGTTTA TCATTGACAC GGAC TCATCAG ACCGGA AACGCA TCCGGT GACAGC CTGGCGAGATATACGGAAAAG AG GC
**BT1442**	W_R6R30c_X-RC	NGS reference	CC TTTTCTTTTT GCTGT TTC GTCC TCGGCATAGC GTATCACCGA TCATTGACAC GGAC TCATCAG ACCGGA AAGTCA TCCGGT GACAGC TTTGTTTGTTT CCC AA GC
**BT1512**	Z_R6R30d_X-RC	NGS reference	CC TTTTCTTTTT GCTGT TTC GTCC TCGGCATAGC TCACCGGAAGTCATTGACAC GGAC TCATCAG ACCGGA AAGTCA TCCGGT GACAGC GTTTTGTTTTGT CCC AC GC
**BT1727**	A_R60R5a_X-RC	NGS reference	CC TTTCTTTTT GCTGT TTC GTCC AGCTA GGAC TCATCAG ACCGGA TCTGTACGCCTAGGTGGCTA ACGACTGTCG ATCGCACATGACGTTGCAAATGTAAGTTCT TCCGGT GACAGC CTGGCGAGATATACGGAAAAG AG GC
**BT1728**	W_R60R5b_X-RC	NGS reference	CC TTTCTTTTT GCTGT TTC GTCC AGCTA GGAC TCATCAG ACCGGA TCTGTACGCCTAGGTGGCTA GAACCGGCTT ATCGCACATGACGTTGCAAATGTAAGTTCT TCCGGT GACAGC TTTGTTTGTTT CCC AA GC
**BT1729**	Z_R60R5c_X-RC	NGS reference	CC TTTCTTTTT GCTGT TTC GTCC AGCTA GGAC TCATCAG ACCGGA TCTGTACGCCTAGGTGGCTA TCTCGCAGGA ATCGCACATGACGTTGCAAATGTAAGTTCT TCCGGT GACAGC GTTTTGTTTTGT CCC AC GC
**BT1730**	A_R7R4a_X-RC	NGS reference	CC TTTCTTTTT GCTGT TTC GTCC TACC GGAC TCATCAG ACCGGA CAGACAA TCCGGT GACAGC CTGGCGAGATATACGGAAAAG AG GC
**BT1731**	W_R7R4b_X-RC	NGS reference	CC TTTCTTTTT GCTGT TTC GTCC TACC GGAC TCATCAG ACCGGA AAAGCCA TCCGGT GACAGC TTTGTTTGTTT CCC AA GC
**BT1732**	Z_R7R4c_X-RC	NGS reference	CC TTTCTTTTT GCTGT TTC GTCC TACC GGAC TCATCAG ACCGGA CGAAAAC TCCGGT GACAGC GTTTTGTTTTGT CCC AC GC
**BT1733**	A_R35R6a_X-RC	NGS reference	CC TTTCTTTTT GCTGT TTC GTCC AACGTA GGAC TCATCAG ACCGGA TCGGCATAGC ACATAGCCGAGTTCG TCATTGACAC TCCGGT GACAGC CTGGCGAGATATACGGAAAAG AG GC
**BT1734**	W_R35R6b_X-RC	NGS reference	CC TTTCTTTTT GCTGT TTC GTCC ACGTAA GGAC TCATCAG ACCGGA TCGGCATAGC CCTCGTATGAACGAG TCATTGACAC TCCGGT GACAGC TTTGTTTGTTT CCC AA GC
**BT1735**	Z_R35R6c_X-RC	NGS reference	CC TTTCTTTTT GCTGT TTC GTCC AGTACA GGAC TCATCAG ACCGGA TCGGCATAGC ACGGTCCGACAATTG TCATTGACAC TCCGGT GACAGC GTTTTGTTTTGT CCC AC GC
**BT1736**	A_R25R6a_X-RC	NGS reference	CC TTTCTTTTT GCTGT TTC GTCC ACTAGA GGAC TCATCAG ACCGGA TCGGCATAGC GCTGA TCATTGACAC TCCGGT GACAGC CTGGCGAGATATACGGAAAAG AG GC
**BT1737**	W_R25R6b_X-RC	NGS reference	CC TTTCTTTTT GCTGT TTC GTCC ACATGA GGAC TCATCAG ACCGGA TCGGCATAGC CTGAG TCATTGACAC TCCGGT GACAGC TTTGTTTGTTT CCC AA GC
**BT1738**	Z_R25R6c_X-RC	NGS reference	CC TTTCTTTTT GCTGT TTC GTCC AGTCAA GGAC TCATCAG ACCGGA TCGGCATAGC TGAGC TCATTGACAC TCCGGT GACAGC GTTTTGTTTTGT CCC AC GC
**BT236p**	Illumina Left Outer	NGS Primer	AATGATACGGCGACCACCGA

**BT235p**	Illumina Right Outer	NGS Primer	CAAGCAGAAGACGGCATACG
**BT1333**	Illumina_Left_W1	NGS Primer	AATGATACGGCGACCACCGAGATCT ACAC TCTTTCCCTACACGACGCTCTTCCGATCT GGG AAACAAACAAA GCTG
**BT1335**	Illumina_Left_W2	NGS Primer	AATGATACGGCGACCACCGAGATCT ACAC TCTTTCCCTACACGACGCTCTTCCGATCT AGAT GGG AAACAAACAAA GCTG
**BT1334**	Illumina_Left_W3	NGS Primer	AATGATACGGCGACCACCGAGATCT ACAC TCTTTCCCTACACGACGCTCTTCCGATCT CC GGG AAACAAACAAA GCTG
**BT1336**	Illumina_Left_W4	NGS Primer	AATGATACGGCGACCACCGAGATCT ACAC TCTTTCCCTACACGACGCTCTTCCGATCT ACGGTT GGG AAACAAACAAA GCTG
**BT1422**	Illumina_Left_W5	NGS Primer	AATGATACGGCGACCACCGAGATCT ACAC TCTTTCCCTACACGACGCTCTTCCGATCT T GGG AAACAAACAAA GCTG
**BT1423**	Illumina_Left_W6	NGS Primer	AATGATACGGCGACCACCGAGATCT ACAC TCTTTCCCTACACGACGCTCTTCCGATCT GAC GGG AAACAAACAAA GCTG
**BT484**	Illumina_Left_A1	NGS Primer	AATGATACGGCGACCACCGAGATCTACACTCTTTCCCTACACGACGCTCTTCCGATCT CTTTTCCGTATATCTCGCCAG
**BT488**	Illumina_Left_A2	NGS Primer	AATGATACGGCGACCACCGAGATCTACACTCTTTCCCTACACGACGCTCTTCCGATCT AGAT CTTTTCCGTATATCTCGCCAG
**BT492**	Illumina_Left_A3	NGS Primer	AATGATACGGCGACCACCGAGATCTACACTCTTTCCCTACACGACGCTCTTCCGATCT CC CTTTTCCGTATATCTCGCCAG
**BT494**	Illumina_Left_A4	NGS Primer	AATGATACGGCGACCACCGAGATCTACACTCTTTCCCTACACGACGCTCTTCCGATCT GTGAA CTTTTCCGTATATCTCGCCAG
**BT490**	Illumina_Left_A5	NGS Primer	AATGATACGGCGACCACCGAGATCTACACTCTTTCCCTACACGACGCTCTTCCGATCT T CTTTTCCGTATATCTCGCCAG
**BT486**	Illumina_Left_A6	NGS Primer	AATGATACGGCGACCACCGAGATCTACACTCTTTCCCTACACGACGCTCTTCCGATCT GAC CTTTTCCGTATATCTCGCCAG
**BT1533**	Illumina_Left_Z1	NGS Primer	AATGATACGGCGACCACCGAGATCT ACAC TCTTTCCCTACACGACGCTCTTCCGATCT GGG ACAAAACAAAAC GC
**BT1534**	Illumina_Left_Z2	NGS Primer	AATGATACGGCGACCACCGAGATCT ACAC TCTTTCCCTACACGACGCTCTTCCGATCT AGAT GGG ACAAAACAAAAC GC
**BT1535**	Illumina_Left_Z3	NGS Primer	AATGATACGGCGACCACCGAGATCT ACAC TCTTTCCCTACACGACGCTCTTCCGATCT CC GGG ACAAAACAAAAC GC
**BT1536**	Illumina_Left_Z4	NGS Primer	AATGATACGGCGACCACCGAGATCT ACAC TCTTTCCCTACACGACGCTCTTCCGATCT ACGGTT GGG ACAAAACAAAAC GC
**BT1537**	Illumina_Left_Z5	NGS Primer	AATGATACGGCGACCACCGAGATCT ACAC TCTTTCCCTACACGACGCTCTTCCGATCT T GGG ACAAAACAAAAC GC
**BT1538**	Illumina_Left_Z6	NGS Primer	AATGATACGGCGACCACCGAGATCT ACAC TCTTTCCCTACACGACGCTCTTCCGATCT GAC GGG ACAAAACAAAAC GC
**BT607**	Illumuna_Right_X1	NGS Primer	CAAGCAGAAGACGGCATACGAGATCGTGATGTGACTGGAGTTCAGACGTGTGCTCTTCCGATCT TTTCTTTTT GCTGTTTCGTC
**BT610**	Illumuna_Right_X2	NGS Primer	CAAGCAGAAGACGGCATACGAGATTGGTCAGTGACTGGAGTTCAGACGTGTGCTCTTCCGATCT A TTTCTTTTT GCTGTTTCGTC
**BT609**	Illumuna_Right_X3	NGS Primer	CAAGCAGAAGACGGCATACGAGATGCCTAAGTGACTGGAGTTCAGACGTGTGCTCTTCCGATCT AA TTTCTTTTT GCTGTTTCGTC
**BT907**	Illumuna_Right_X4	NGS Primer	CAAGCAGAAGACGGCATACGAGATATTGGCGTGACTGGAGTTCAGACGTGTGCTCTTCCGATCT CCG TTTCTTTTT GCTGTTTCGTC
**BT908**	Illumuna_Right_X5	NGS Primer	CAAGCAGAAGACGGCATACGAGATGATCTGGTGACTGGAGTTCAGACGTGTGCTCTTCCGATCT GCGC TTTCTTTTT GCTGTTTCGTC
**BT909**	Illumuna_Right_X6	NGS Primer	CAAGCAGAAGACGGCATACGAGATAAGCTAGTGACTGGAGTTCAGACGTGTGCTCTTCCGATCT TGCACA TTTCTTTTT GCTGTTTCGTC
**BT910**	Illumuna_Right_X7	NGS Primer	CAAGCAGAAGACGGCATACGAGATTACAAGGTGACTGGAGTTCAGACGTGTGCTCTTCCGATCT GGCTACC TTTCTTTTT GCTGTTTCGTC
**BT1275**	Illumuna_Right_X8	NGS Primer	CAAGCAGAAGACGGCATACGAGATTTGACTGTGACTGGAGTTCAGACGTGTGCTCTTCCGATCT CAAGGGAA TTTCTTTTT GCTGTTTCGTC
**BT1460**	Illumuna_Right_X10	NGS Primer	CAAGCAGAAGACGGCATACGAGATGGAACTGTGACTGGAGTTCAGACGTGTGCTCTTCCGATCT GACTG TTTCTTTTT GCTGTTTCGTC
**BT1461**	Illumuna_Right_X11	NGS Primer	CAAGCAGAAGACGGCATACGAGATTGACATGTGACTGGAGTTCAGACGTGTGCTCTTCCGATCT GA TTTCTTTTT GCTGTTTCGTC
**BT1462**	Illumuna_Right_X12	NGS Primer	CAAGCAGAAGACGGCATACGAGATGGACGGGTGACTGGAGTTCAGACGTGTGCTCTTCCGATCT ACT TTTCTTTTT GCTGTTTCGTC
**BT1165p**	T7_W_N4_N60_X-RC	Selection Library	TTTTTATTTTTCTTTTT GCTGT TTC GTCC NNNNNNNNNN NNNNNNNNNN NNNNNNNNNN NNNNNNNNNN NNNNNNNNNN NNNNNNNNNN GGAC TCATCAG ACCGGA NNNN TCCGGT GACAGC TTTGTTTGTTT CCCTATAGTGAGTCGTATTAAATT
**BT1166p**	T7_W_N5_N60_X-RC	Selection Library	TTTTTATTTTTCTTTTT GCTGT TTC GTCC NNNNNNNNNN NNNNNNNNNN NNNNNNNNNN NNNNNNNNNN NNNNNNNNNN NNNNNNNNNN GGAC TCATCAG ACCGGA NNNNN TCCGGT GACAGC TTTGTTTGTTT CCCTATAGTGAGTCGTATTAAATT
**BT1167p**	T7_W_N6_N60_X-RC	Selection Library	TTTTTATTTTTCTTTTT GCTGT TTC GTCC NNNNNNNNNN NNNNNNNNNN NNNNNNNNNN NNNNNNNNNN NNNNNNNNNN NNNNNNNNNN GGAC TCATCAG ACCGGA NNNNNN TCCGGT GACAGC TTTGTTTGTTT CCCTATAGTGAGTCGTATTAAATT
**BT1168p**	T7_W_N7_N60_X-RC	Selection Library	TTTTTATTTTTCTTTTT GCTGT TTC GTCC NNNNNNNNNN NNNNNNNNNN NNNNNNNNNN NNNNNNNNNN NNNNNNNNNN NNNNNNNNNN GGAC TCATCAG ACCGGA NNNNNNN TCCGGT GACAGC TTTGTTTGTTT CCCTATAGTGAGTCGTATTAAATT
**BT1169p**	T7_W_N8_N60_X-RC	Selection Library	TTTTTATTTTTCTTTTT GCTGT TTC GTCC NNNNNNNNNN NNNNNNNNNN NNNNNNNNNN NNNNNNNNNN NNNNNNNNNN NNNNNNNNNN GGAC TCATCAG ACCGGA NNNNNNNN TCCGGT GACAGC TTTGTTTGTTT CCCTATAGTGAGTCGTATTAAATT

**BT1171p**	T7_W_N60_N4_X-RC	Selection Library	TTTTTATTTTTCTTTTT GCTGT TTC GTCC NNNN GGAC TCATCAG ACCGGA NNNNNNNNNN NNNNNNNNNN NNNNNNNNNN NNNNNNNNNN NNNNNNNNNN NNNNNNNNNN TCCGGT GACAGC TTTGTTTGTTT CCCTATAGTGAGTCGTATTAAATT
**BT1172p**	T7_W_N60_N5_X-RC	Selection Library	TTTTTATTTTTCTTTTT GCTGT TTC GTCC NNNNN GGAC TCATCAG ACCGGA NNNNNNNNNN NNNNNNNNNN NNNNNNNNNN NNNNNNNNNN NNNNNNNNNN NNNNNNNNNN TCCGGT GACAGC TTTGTTTGTTT CCCTATAGTGAGTCGTATTAAATT
**BT1173p**	T7_W_N60_N6_X-RC	Selection Library	TTTTTATTTTTCTTTTT GCTGT TTC GTCC NNNNNN GGAC TCATCAG ACCGGA NNNNNNNNNN NNNNNNNNNN NNNNNNNNNN NNNNNNNNNN NNNNNNNNNN NNNNNNNNNN TCCGGT GACAGC TTTGTTTGTTT CCCTATAGTGAGTCGTATTAAATT
**BT1174p**	T7_W_N60_N7_X-RC	Selection Library	TTTTTATTTTTCTTTTT GCTGT TTC GTCC NNNNNNN GGAC TCATCAG ACCGGA NNNNNNNNNN NNNNNNNNNN NNNNNNNNNN NNNNNNNNNN NNNNNNNNNN NNNNNNNNNN TCCGGT GACAGC TTTGTTTGTTT CCCTATAGTGAGTCGTATTAAATT
**BT1175p**	T7_W_N60_N8_X-RC	Selection Library	TTTTTATTTTTCTTTTT GCTGT TTC GTCC NNNNNNNN GGAC TCATCAG ACCGGA NNNNNNNNNN NNNNNNNNNN NNNNNNNNNN NNNNNNNNNN NNNNNNNNNN NNNNNNNNNN TCCGGT GACAGC TTTGTTTGTTT CCCTATAGTGAGTCGTATTAAATT
**BT1321p**	T7_W_N4_N30_X-RC	Selection Library	TTTTTATTTTTCTTTTT GCTGT TTC GTCC NNNNNNNNNN NNNNNNNNNN NNNNNNNNNN GGAC TCATCAG ACCGGA NNNN TCCGGT GACAGC TTTGTTTGTTT CCCTATAGTGAGTCGTATTAAATT
**BT1322p**	T7_W_N5_N30_X-RC	Selection Library	TTTTTATTTTTCTTTTT GCTGT TTC GTCC NNNNNNNNNN NNNNNNNNNN NNNNNNNNNN GGAC TCATCAG ACCGGA NNNNN TCCGGT GACAGC TTTGTTTGTTT CCCTATAGTGAGTCGTATTAAATT
**BT1323p**	T7_W_N6_N30_X-RC	Selection Library	TTTTTATTTTTCTTTTT GCTGT TTC GTCC NNNNNNNNNN NNNNNNNNNN NNNNNNNNNN GGAC TCATCAG ACCGGA NNNNNN TCCGGT GACAGC TTTGTTTGTTT CCCTATAGTGAGTCGTATTAAATT
**BT1324p**	T7_W_N7_N30_X-RC	Selection Library	TTTTTATTTTTCTTTTT GCTGT TTC GTCC NNNNNNNNNN NNNNNNNNNN NNNNNNNNNN GGAC TCATCAG ACCGGA NNNNNNN TCCGGT GACAGC TTTGTTTGTTT CCCTATAGTGAGTCGTATTAAATT
**BT1325p**	T7_W_N8_N30_X-RC	Selection Library	TTTTTATTTTTCTTTTT GCTGT TTC GTCC NNNNNNNNNN NNNNNNNNNN NNNNNNNNNN GGAC TCATCAG ACCGGA NNNNNNNN TCCGGT GACAGC TTTGTTTGTTT CCCTATAGTGAGTCGTATTAAATT
**BT1326p**	T7_W_N30_N4_X-RC	Selection Library	TTTTTATTTTTCTTTTT GCTGT TTC GTCC NNNN GGAC TCATCAG ACCGGA NNNNNNNNNN NNNNNNNNNN NNNNNNNNNN TCCGGT GACAGC TTTGTTTGTTT CCCTATAGTGAGTCGTATTAAATT
**BT1327p**	T7_W_N30_N5_X-RC	Selection Library	TTTTTATTTTTCTTTTT GCTGT TTC GTCC NNNNN GGAC TCATCAG ACCGGA NNNNNNNNNN NNNNNNNNNN NNNNNNNNNN TCCGGT GACAGC TTTGTTTGTTT CCCTATAGTGAGTCGTATTAAATT
**BT1328p**	T7_W_N30_N6_X-RC	Selection Library	TTTTTATTTTTCTTTTT GCTGT TTC GTCC NNNNNN GGAC TCATCAG ACCGGA NNNNNNNNNN NNNNNNNNNN NNNNNNNNNN TCCGGT GACAGC TTTGTTTGTTT CCCTATAGTGAGTCGTATTAAATT
**BT1329p**	T7_W_N30_N7_X-RC	Selection Library	TTTTTATTTTTCTTTTT GCTGT TTC GTCC NNNNNNN GGAC TCATCAG ACCGGA NNNNNNNNNN NNNNNNNNNN NNNNNNNNNN TCCGGT GACAGC TTTGTTTGTTT CCCTATAGTGAGTCGTATTAAATT
**BT1330p**	T7_W_N30_N8_X-RC	Selection Library	TTTTTATTTTTCTTTTT GCTGT TTC GTCC NNNNNNNN GGAC TCATCAG ACCGGA NNNNNNNNNN NNNNNNNNNN NNNNNNNNNN TCCGGT GACAGC TTTGTTTGTTT CCCTATAGTGAGTCGTATTAAATT
**JX457**	X_G12A_polyA-RC	Reverse primer to mutate G12A for SPR	TTTTTTTTTTTTTTTTTTTTTTTT GCTGTTTTGTC
**mJX1**	SPR_Binding_FWD	Forward primer for minimal aptamer SPR	TTCTAATACGACTCACTATAGGG
**mJX2**	SPR_Binding_REV	Reverse primer for minimal aptamer for SPR	TTTTTTTTTTTTTTTTTTTTTTTTGGGG
**sJX105**	pCS1748_fwd	Forward primer for Gibson cloning swtiches into yeast expression plasmid pCS1748	TCCATGGTATGGATGAATTGTACAAATAAAGCCTAGGAAACAAACAAAGCTGTCAC
**sJX18**	pCS1748_rev	Reverse primer for Gibson cloning swtiches into yeast expression plasmid pCS1748	AAGAAATTCGCTTATTTAGAAGTGGCGCGCCCTCTCGAGTTTTTATTTTT CTTTTTGCTGTTTCG
**JX562**	T7_zea579_loopI_polyA_RC	SPR template	TTTTTTTTTTTTTTTTTTTTTTTTGGGGACCGGAGGCGCTTCTACGCACTTGCATTCTGTTCTACCC GGTCCCTATAGTGAGTCGTATTAGAA
**JX563**	T7_nosc441_polyA_left	SPR template	TTCTAATACGACTCACTATAGGGACCGGAAGAACTGACATTTGCAACGTCATGTGCGATGTCCAACG TGTAGCCACCTAGGC
**JX564**	nosc441_polyA_right-RC	SPR template	TTTTTTTTTTTTTTTTTTTTTTTTGGGGACCGGATCTGTACGCCTAGGTGGCTACACGTT
**JX565**	T7_acic145_polyA_RC	SPR template	TTTTTTTTTTTTTTTTTTTTTTTTGGGGACCGGATTTCGGTAGCCCATAAACTACTTCGGTTATTCC GGTCCCTATAGTGAGTCGTATTAGAA
**JX566**	T7_zea927_polyA_RC	SPR template	TTTTTTTTTTTTTTTTTTTTTTTTGGGGGTCCTGTCCACGTATCACACTGGGTCGCATGGAGGGACC CCTATAGTGAGTCGTATTAGAA
**JX567**	T7_theo421_polyA_MFE_RC	SPR template	TTTTTTTTTTTTTTTTTTTTTTTTGGGGGTCCTGAGTATCGTACCGCTGGAAGGCACATGAGGGACC CCTATAGTGAGTCGTATTAGAA
**JX568**	T7_theo421_polyA_subopt1_RC	SPR template	TTTTTTTTTTTTTTTTTTTTTTTTGGGGTGAGTATCGTACCGCTGGAAGGCACATGAGGGACTCACC CTATAGTGAGTCGTATTAGAA
**JX569**	T7_sret760_N5_G12A	SPR template	GGG AAACAAACAAA GCTGTCACTGGA NNNNN TCCAGTCTGATGAGTCC TATAGGCACGCTTCCCCCAGGTGTAGGGA GGACAAAACAGC AAAAAGAAA
**JX572**	T7_SRet760_polyA_RC	SPR template	TTTTTTTTTTTTTTTTTTTTTTTTGGGGGTCCTCCCTACACCTGGGGGAAGCGTGCCTATAGGACCC CTATAGTGAGTCGTATTAGAA
**JX573**	N5_zea927_G12A	SPR template	GGGAAACAAACAAA GCTGTC ACCGGA NNNNN TCTGGT CTGATGA GTCC CTCCATGCGACCCAGTGTGATACGTGGACA GGACAAAACAGC AAAAAGAAA

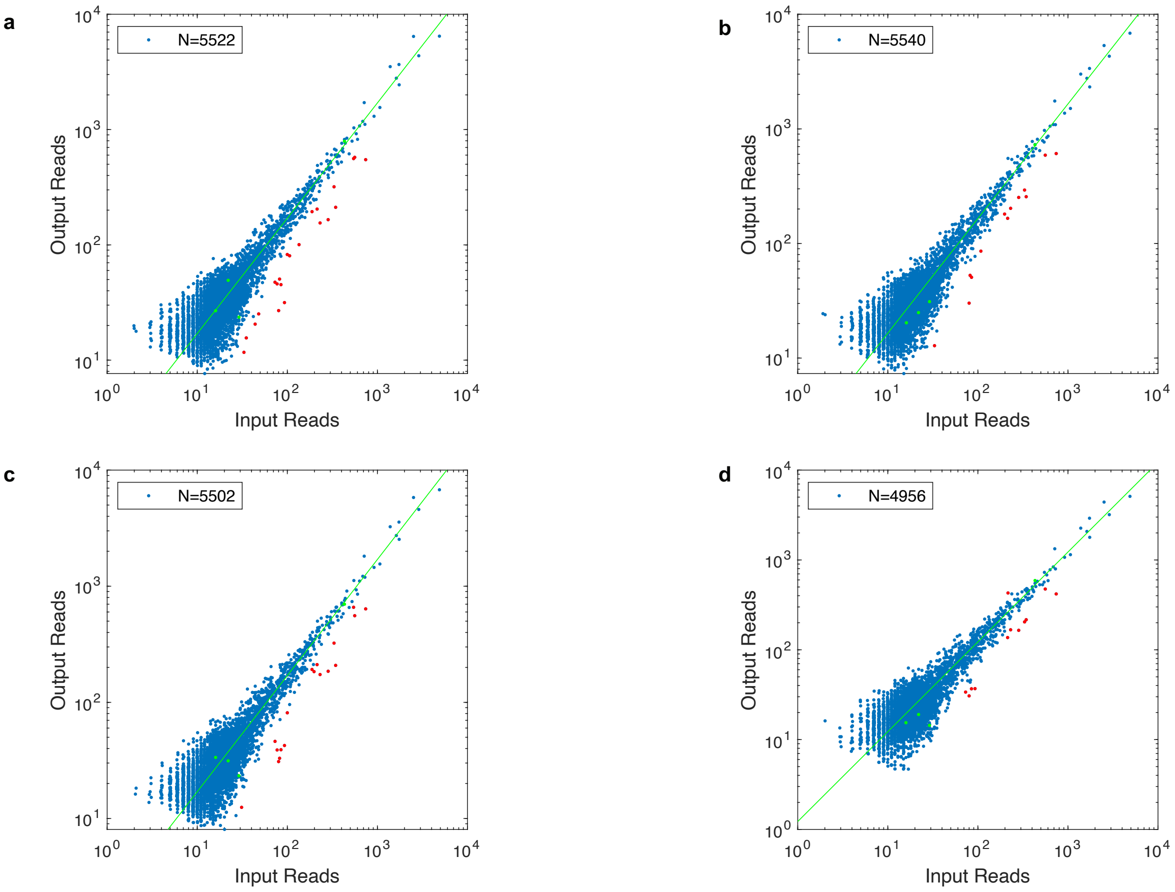

Supplementary Figure 1 | Efficiency of CleaveSeq including regeneration. CleaveSeq was run on a library of biosensor sequences formed from mixing the products of rounds 91, 98, 106, 114, and 122 of DRIVER select S3 and then constricting to ~5,000 distinct sequences. The total number of reads attributed to cleaved and uncleaved products (each normalized using reference sequences) for each sequence is plotted against the number of reads of the same sequences at the input to CleaveSeq. The green line shows the expected output reads based on the ratio of total NGS reads allocated to the input and output libraries. Red points indicate sequences with output counts significantly lower than other sequences based on a two-sided two-proportion z-test with $p<0.01$ and applying a Bonferroni correction for multiple hypotheses. **a.** CleaveSeq with no ligand present, **b.** T1 ligand mixture, **c.** T2e ligand mixture, **d.** T3e ligand mixture. Source data are provided as a Source Data file.

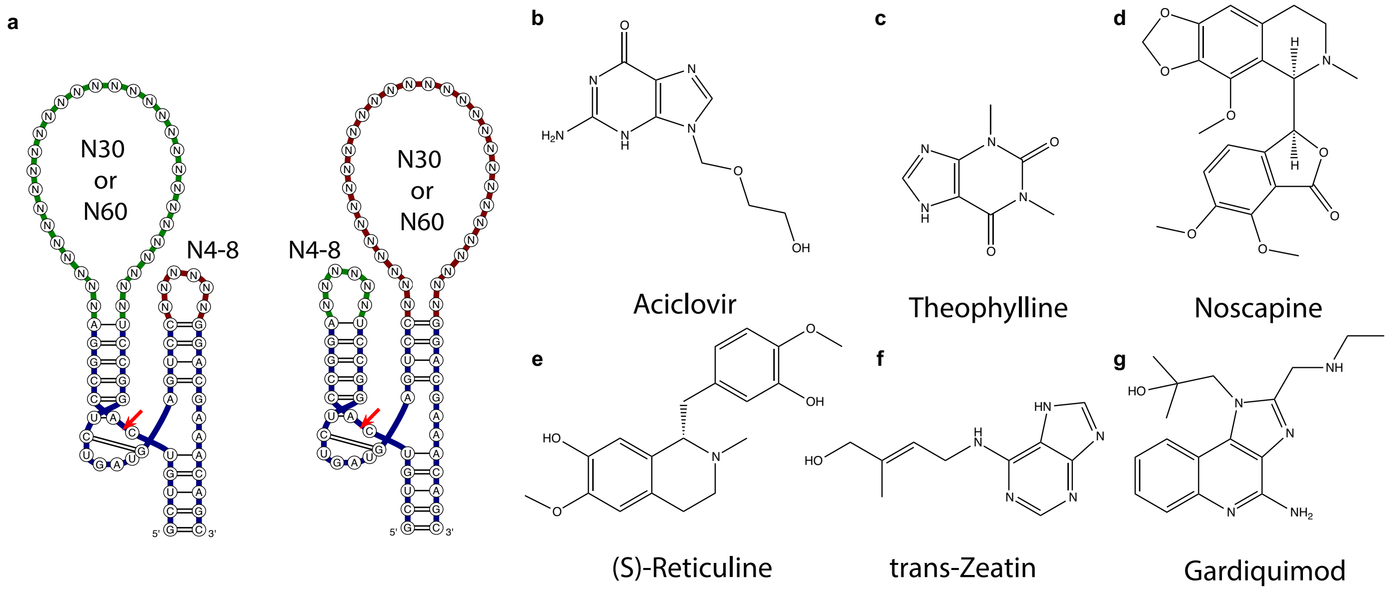

Supplementary Figure 2 | Library design and ligand structures. a. Secondary structure representation of general biosensor library design with the loop randomizations indicated. N6 small loops and N30 large loops are shown. **b-g.** Chemical structures of ligands for which novel biosensors were validated in this work: aciclovir (**b),** theophylline (**c**), noscapine (**d),** (*S)*-reticuline (**e),** trans-zeatin (**f**), gardiquimod (**g**).

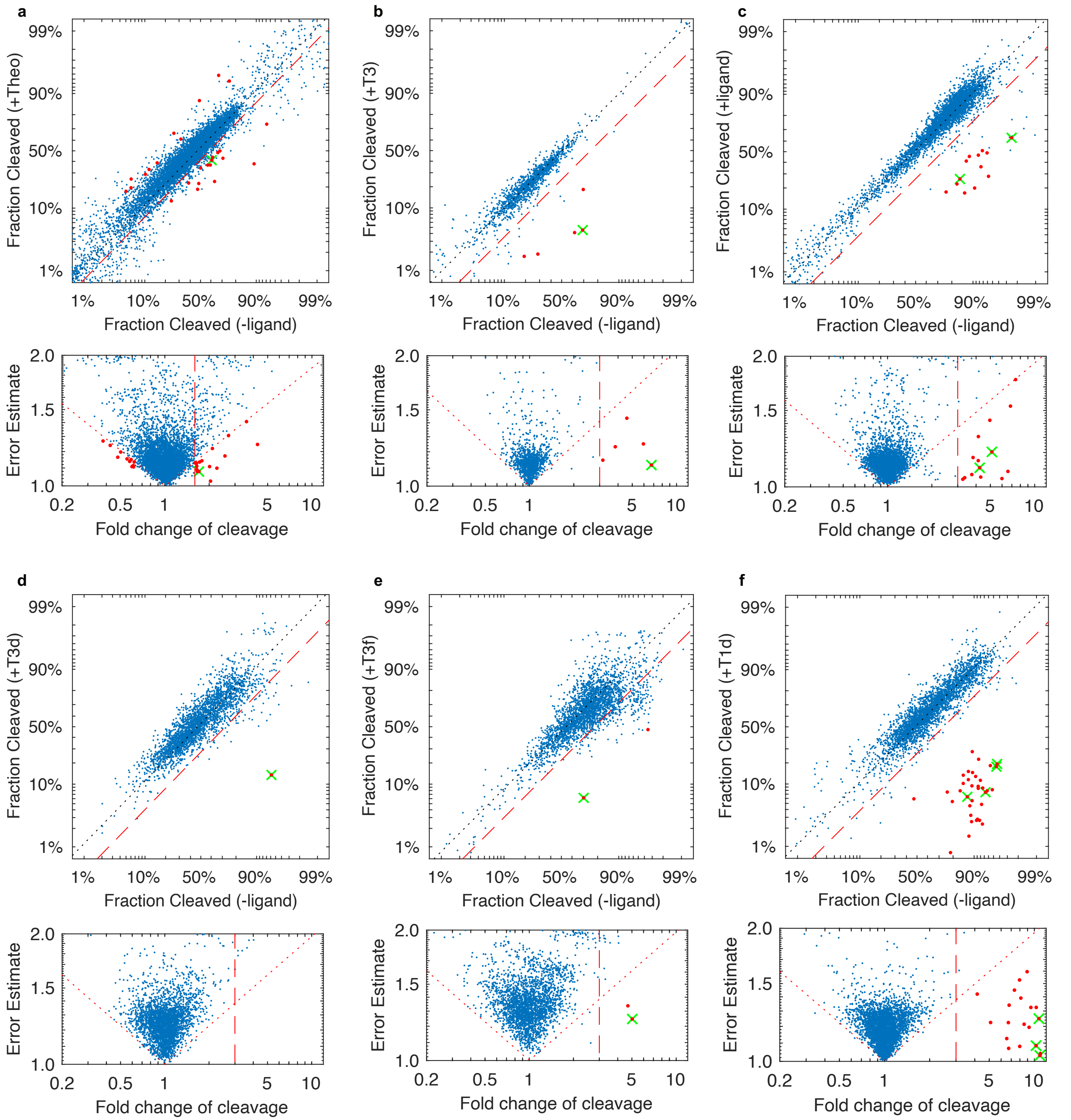

Supplementary Figure 3 | Fraction cleaved and fold change of fraction cleaved for evolved libraries. Comparison of cleavage fractions in the presence and absence of the ligand mixture determined via CleaveSeq of libraries at various points during the selections. In each section, the top subpanel shows the fraction cleaved of each of the sequences which have at least 30 reads in each of the -ligand and +ligand conditions. Bottom panel, the same data plotted with the ratio of the cleavage fractions in the presence and absence of the ligand mixture on the x-axis and the standard error of the ratio on the y-axis. Dotted diagonal line, delineates the region where a multiple-hypothesis test would reject the null hypothesis of non-switching, with alpha=1/N. In both panels: dashed line, boundary where the fold change of cleavage is at least 3x (or 2x for theophylline); red dots, indicate sequences with strong, significant (i.e., below the diagonal line and to the right of the dashed line) switching; green crosses, indicate validated biosensors that were first identified from the particular analysis. **a.** S1 round 57 against theophylline (Theo-421 in green). **b.** S2 round 36 against T3 ligand group (SRet-584 in green). **c.** S3 round 74 against T1b ligand group (Acic-711 and Acic-758 in green). **d.** S3 round 84 against T3d ligand group (Nosc-786 in green). **e.** S3 round 150 against T3f ligand group (TZea-927 in green). **f.** S4 round 102 against T1d ligand group (Gard-337, Gard-910, Gard-544, and Gard-674 in green). Source data are provided as a Source Data file.

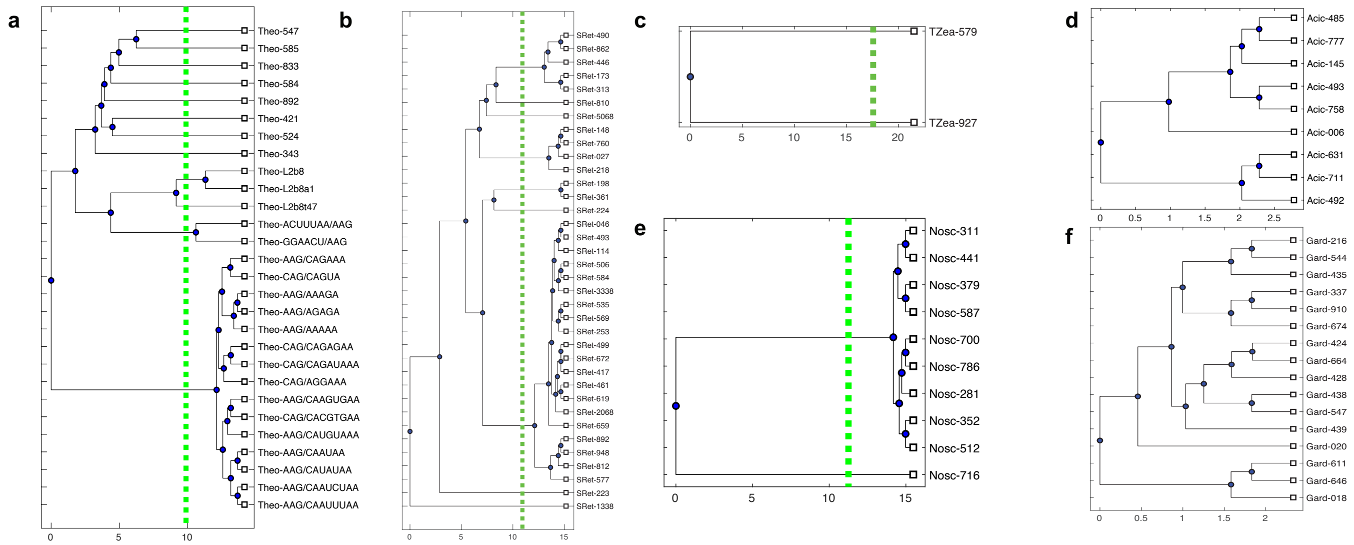

Supplementary Figure 4 | Dendrograms of validated biosensors. Dendrograms of biosensors found for each distinct ligand based on pairwise distance (number of mismatches) with average linkage. A distance of 5 mismatches (green dotted lines) was used to classify groups of sequences into different families. Dendrograms are provided as follows: **a.** theophylline (8 distinct families found during selection S1). The bottom 20 sensors were previously designed biosensors based on the original TCT8-3 theophylline aptamer), **b.** (S)-reticuline (9 distinct families), **c.** trans-zeatin (2 distinct families), **d.** aciclovir (1 family), **e.** noscapine (2 distinct families), **f.** gardiquimod (1 family).

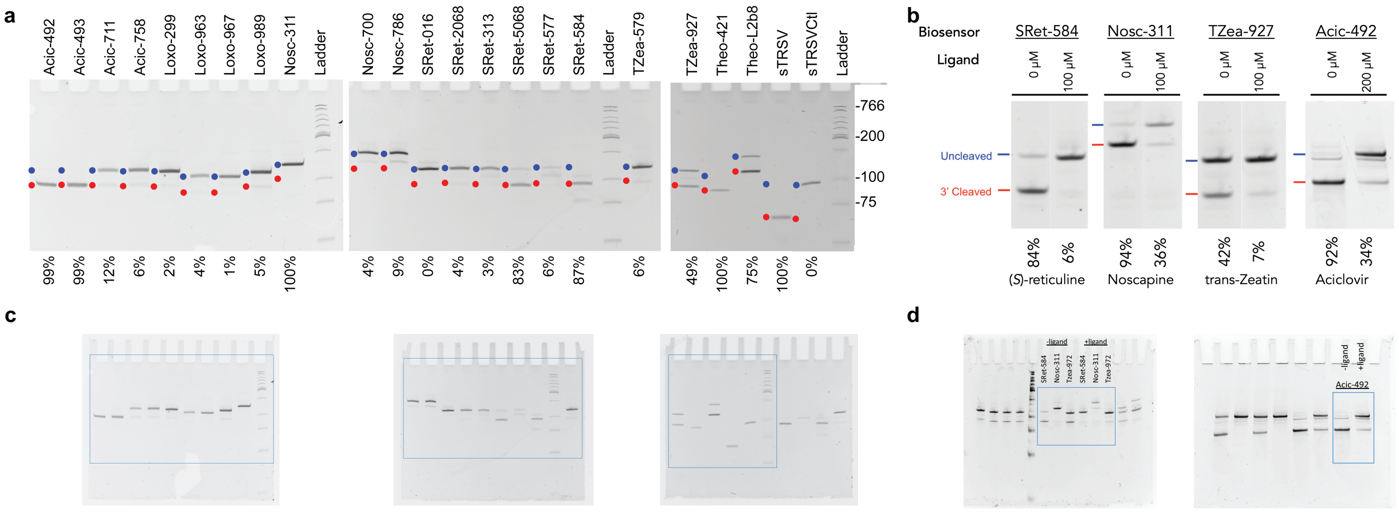

Supplementary Figure 5 | Polyacrylamide gel electrophoresis analysis of RNA biosensors. a. PAGE gel and analysis of biosensors and controls used to determine the fraction cleaved following a co-transcriptional cleavage assay in the absence of ligand. The labels above indicate the particular biosensor or control RNA sequence (Supplementary Data 3), blue and red dots indicate the expected position of the uncleaved and 3'-cleaved products, respectively; numbers below are the ratio of the cleaved band intensity to the total of the cleaved and 3'-uncleaved bands. **b.** PAGE assay of select biosensors showing fraction cleaved in the -ligand and +ligand conditions. **c.** Uncropped gels corresponding to **a**. **d.** Uncropped gels corresponding to **b**. Each PAGE gel electrophoresis experiment was performed once.

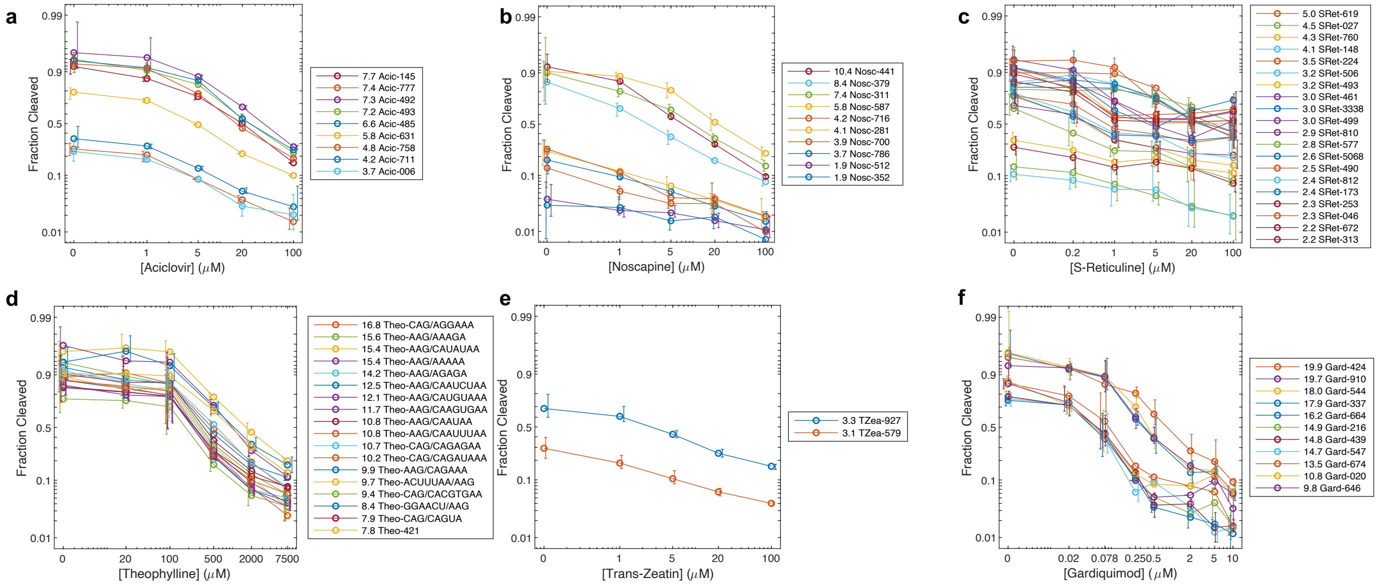

Supplementary Figure 6 | Fraction cleaved for select biosensors as a function of ligand concentration. a-f. The fraction cleaved of DRIVER-selected biosensors was measured over a range of ligand concentrations using the CleaveSeq assay. Each plot shows the biosensors that exhibit a fold change of fraction cleaved of at least 2.0. Points shows measurements derived from at least 100 NGS reads. Legends include the fold change of fraction cleaved of the indicated biosensor over the ligand range tested. Error bars are [25%,75%] confidence intervals of the mean, calculated over at least n=3 biologically independent experiments; note that error bars are slightly offset from data points to improve legibility. Data is shown for sensors responsive to aciclovir (**a)**, noscapine (**b)**, (*S*)-reticuline (**c)**, theophylline (**d)**, trans-zeatin (**e**), and gardiquimod (**f**). Source data are provided as a Source Data file.

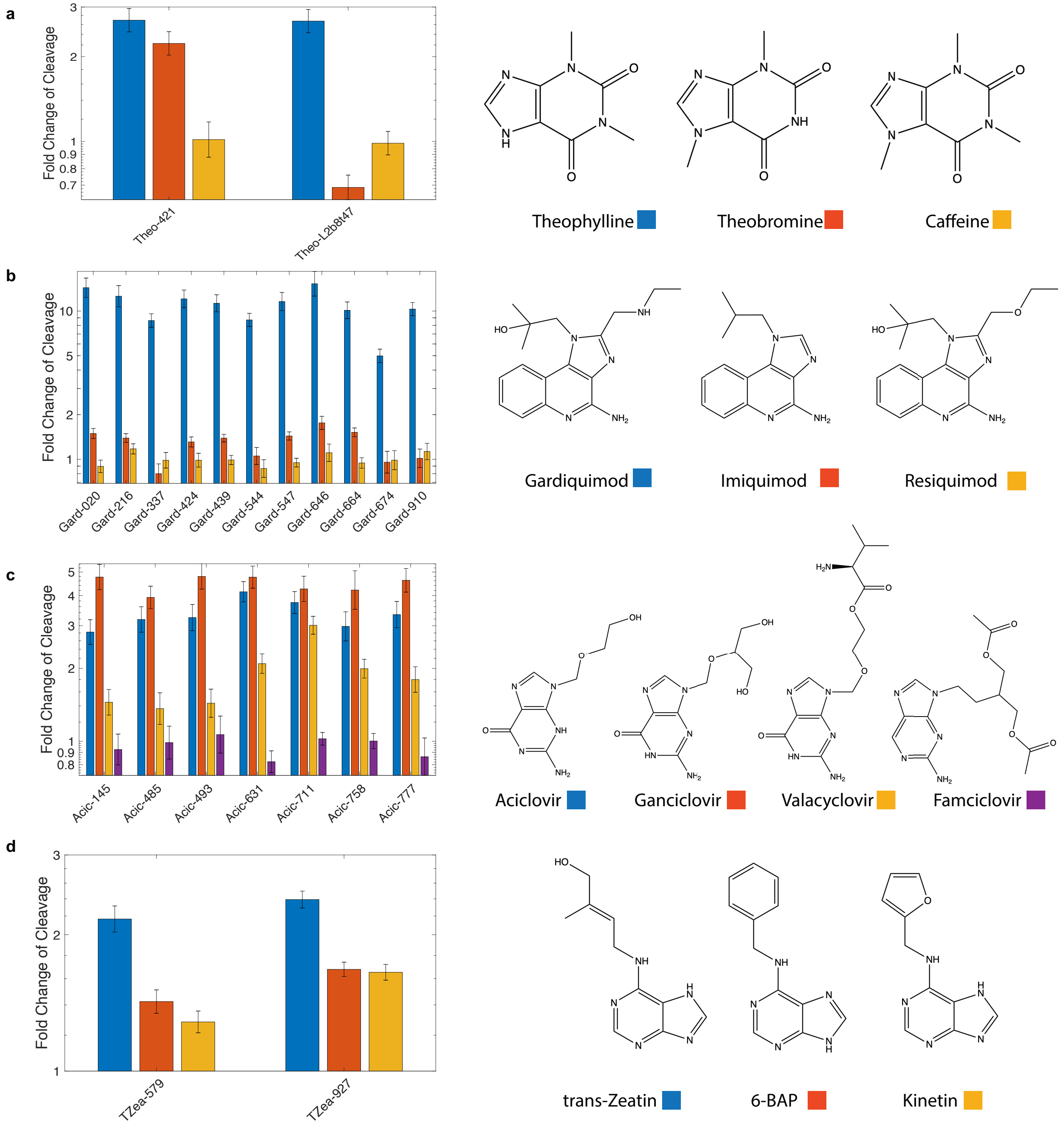
a
Fold Change of Cleavage
Theo-421
Theo-L2b8t47
Theophylline
Theobromine
Caffeine
b
Fold Change of Cleavage
Gard-020
Gard-216
Gard-337
Gard-424
Gard-439
Gard-544
Gard-547
Gard-646
Gard-664
Gard-674
Gard-910
Gardiquimod
Imiquimod
Resiquimod
c
Fold Change of Cleavage
Acic-145
Acic-485
Acic-493
Acic-631
Acic-711
Acic-758
Acic-777
Aciclovir
Ganciclovir
Valacyclovir
Famciclovir
d
Fold Change of Cleavage
TZea-579
TZea-927
trans-Zeatin
6-BAP
Kinetin

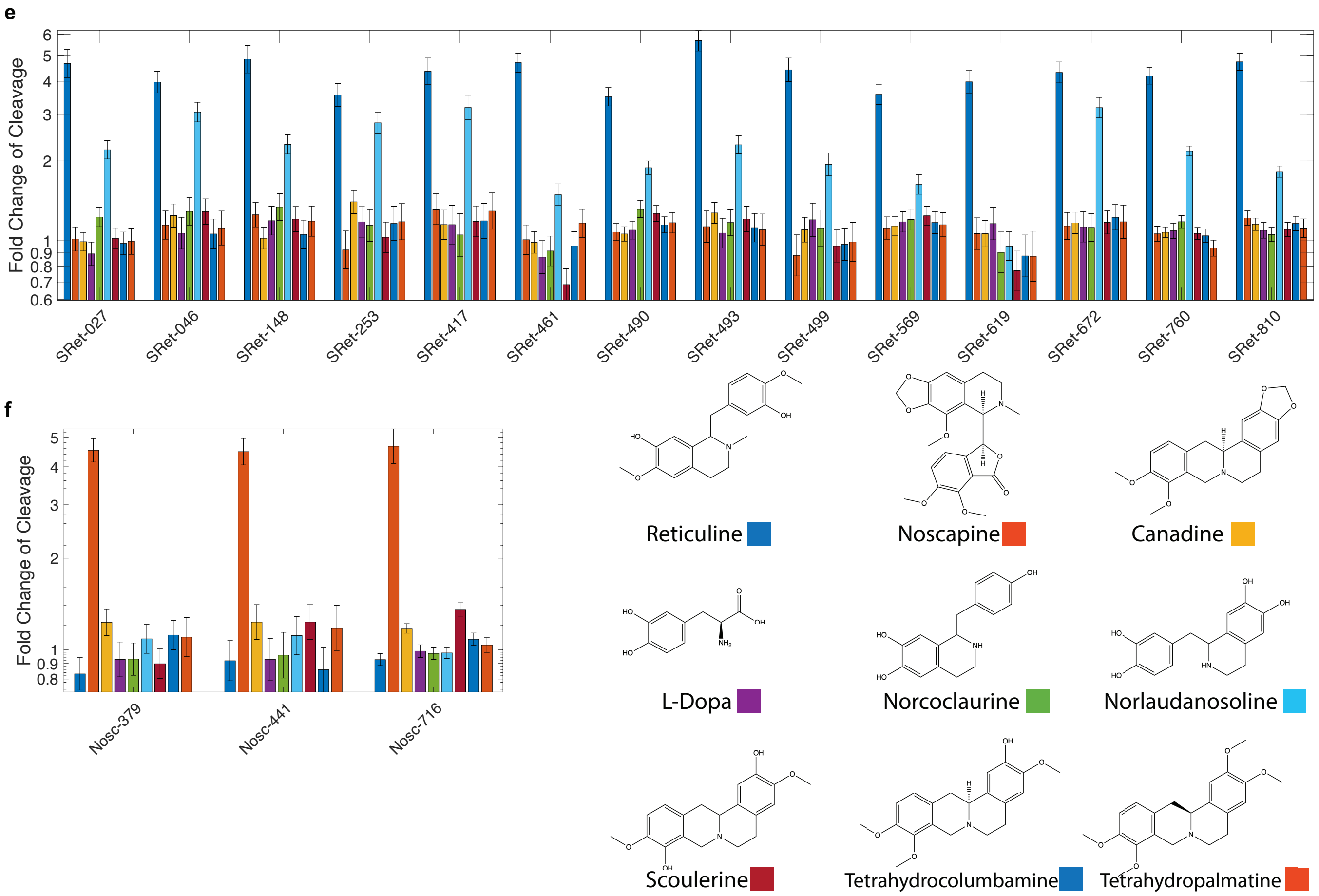

Supplementary Figure 7 | Selectivity of DRIVER Biosensors. a-f. CleaveSeq was used to measure fold change of cleavage of representative biosensors in the presence of their designated targets and several similar molecules relative to the cleavage in the absence of target. For each condition data are presented as the maximum likelihood estimator +/- standard deviation of the fold change of cleavage computed from the statistics of the NGS read counts. **a.** Aciclovir sensors against aciclovir, ganciclovir, valacyclovir, and famciclovir at 20μM. **b.** Theophylline sensors against theophylline, theobromine, and caffeine at 500μM. Theo-L2b8t47 is a previously published sensor based on the TCT8-4 aptamer. **c.** Gardiquimod sensors against gardiquimod, imiquimod, and resiquimod at 20μM. **d.** Trans-zeatin sensor against trans-zeatin, 6-benzylaminopurine, and kinetin at 20μM. **e.** Noscapine sensors against several benzylisoquinoline alkaloids (BIAs) and precursors to BIAs: (*S*)-reticuline, norlaudanosoline, noscapine, canadine, L-dopa, norcoclaurine, scoulerine, tetrahydrocolumbamine, and tetrahydropalmatine at 20μM. **f.** (*S*)-reticuline sensors against the same set of BIAs and precursor BIAs at 20μM. Source data are provided as a Source Data file.

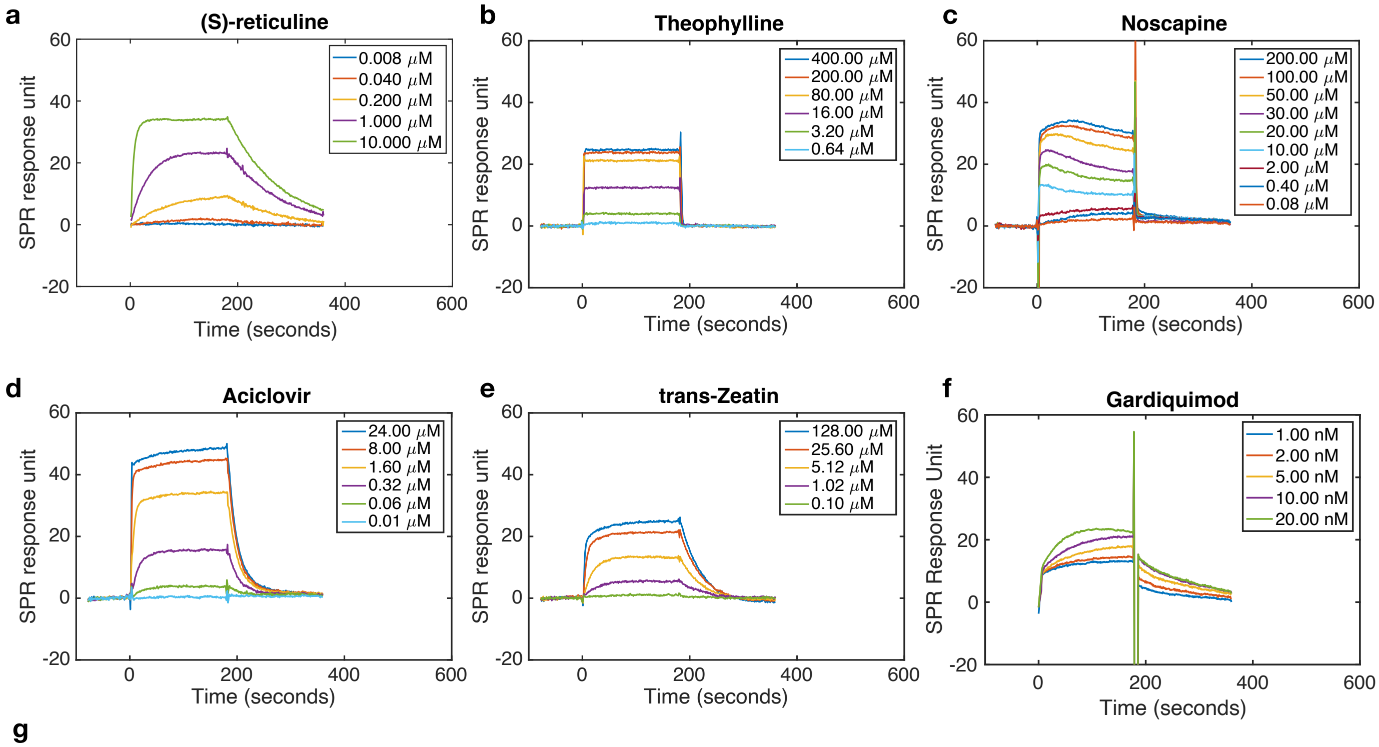

g

	Biosensor	k_{on} ($M^{-1}s^{-1}$)	k_{off} (s^{-1})	Kinetic K_D (M)	Equilibrium K_D (M)
a	SRet-584	1.95E+04	1.00E-02	5.14E-07	4.65E-07
b	Theo-421		NM		1.57E-05
c	Nosc-786		NM		2.87E-05
d	Acic-145	6.85E+04	5.41E-02	7.89E-07	7.04E-07
e	TZea-579	3.42E+03	2.77E-02	8.09E-06	1.13E-05
f	Gard-547		NM		8.13E-09

Supplementary Figure 8 | Surface plasmon resonance (SPR) assay sensorgrams to measure the binding affinity of representative biosensors. **a.-e.** Select biosensors characterized using Surface Plasmon Resonance from a single Biacore run. Representative sensorgrams are provided for the following biosensors: **a.** (*S*)-reticuline biosensor SRet-584, **b.** theophylline biosensor Theo-421, **c.** noscapine biosensor Nosc-786, **d.** aciclovir biosensor Acic-145, **e.** trans-zeatin biosensor TZea-579 and, **f.** gardiquimod biosensor Gard-547. **g.** Table showing binding parameters for the measurable parameters of the sensorgrams in a-f.

a

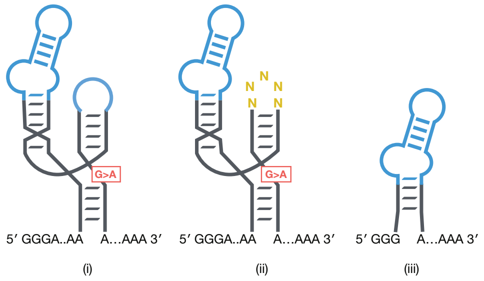

b

Sensor	K_D (μM)		
	**(i)**	**(ii)**	**(iii)**
**Acic-145**	1.5 ± 0.3	0.680 ± 0.004	0.700 ± 0.004
**Nosc-441**	13 ± 2	20 ± 1	20 ± 7
**SRet-760**	0.15 ± 0.01	NB	NB
**Theo-421**	68 ± 20	19 ± 4	NB/17.0 ± 0.7*
**TZea-579**	3.1 ± 0.5	5.6 ± 0.4	37 ± 3
**TZea-927**	62 ± 20	NB	NB
**Gard-547**	0.010	NA	NA

c

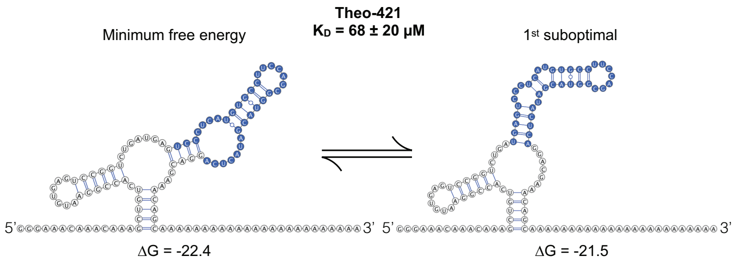

d

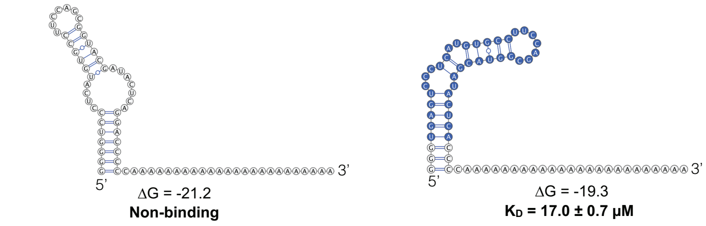

Supplementary Figure 9 | SPR binding affinities of select biosensors and characterization of putative binding domains. a. Schematic showing the modifications made to the sequences of biosensors used in surface plasmon resonance (SPR) binding characterizations; (i) G12A mutation to prevent ribozyme self-cleavage during binding affinity experiments, (ii) randomizing the smaller loop of the biosensor sequence in addition to a G12A mutation, (iii) truncation to include only the stem I or stem II sequences, whichever contains the larger loop, excluding the catalytic core and the rest of the ribozyme context. **b.** Table shows the equilibrium dissociation constants (K_D) as a measure of binding affinity for 6 representative biosensors from five ligand classes (aciclovir, noscapine, (*S*)-reticuline, theophylline, and trans-zeatin) of DRIVER-selected biosensors in the three architectural contexts as illustrated in **a**. Values are mean ± s.e.m of three or more replicate samples. NB, no observed binding. NA, not available. *Theo-421 stem-only (iii) version did not exhibit binding, even though all sequences with preserved binding in (ii) showed binding in (iii); however, the stem-only sequence that stabilizes the first suboptimal structure did, as shown **d**. **c**. Predicted secondary structures of Theo-421 show the minimum free energy structure (top left) and a suboptimal structure (top right), with the predicted free energies ΔG indicated below each structure using the secondary structure folding program RNAstructure. **d.** The truncated structures derived from each of the two structural conformations are shown. K_D corresponds to that in **b**(iii), annotating the structures for clarity. Blue, sequence bases in the putative binding domain.

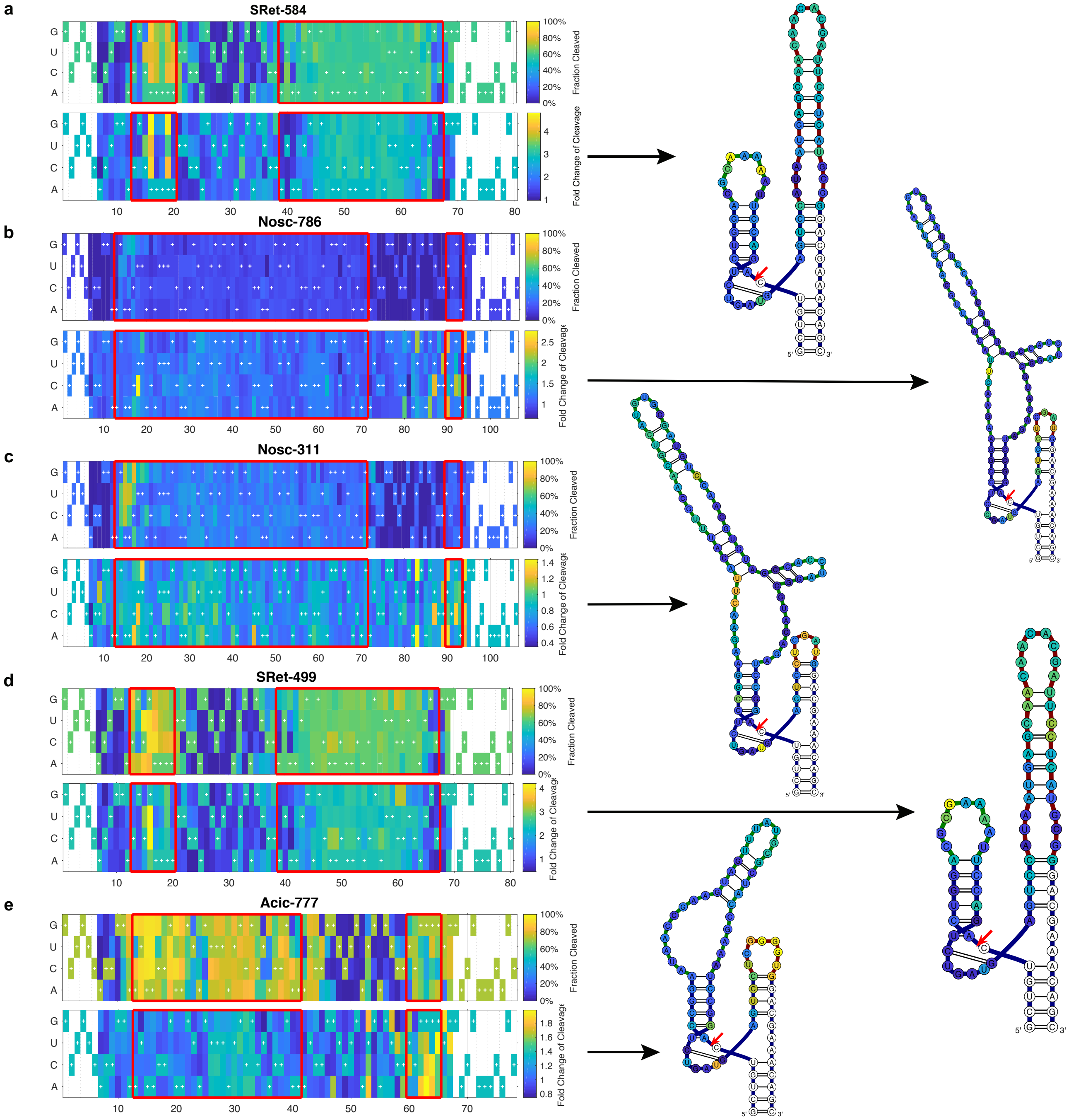

Supplementary Figure 10 | Mutational analyses via CleaveSeq show the effect of single-base mutations to biosensor activity. Each biosensor was mutagenized and the resulting library characterized via CleaveSeq in the presence and absence of ligand. The top left subpanel of each section shows the fraction cleaved as a function of base identity and position in the biosensor without ligand present. Red boxes delineate the loop I and loop II regions of the biosensor; plus symbols indicate the sequence of the wild-type biosensor. The white regions have no mutation data as they overlapped with primers during subsequent PCR. The lower left subpanels show the fold change of fraction cleaved in the presence and absence of the ligand as a function of base identity and position of point mutations in the biosensor. To the right of each heat map, the fold change of cleavage of the most favorable mutation at each nucleotide position was mapped to the secondary structure of the unmutated biosensor. The color mapping is the same as for the corresponding lower left subpanel. **a.** SRet-584 with (*S*)-reticuline at 200 nM, **b.** Nosc-786 with noscapine at 20 μM, **c.** Nosc-311 with noscapine at 5 μM, **d.** SRet-499 with (*S*)-reticuline at 5 μM, **e.** Acic-777 with aciclovir at 5 μM. Source data are provided as a Source Data file.

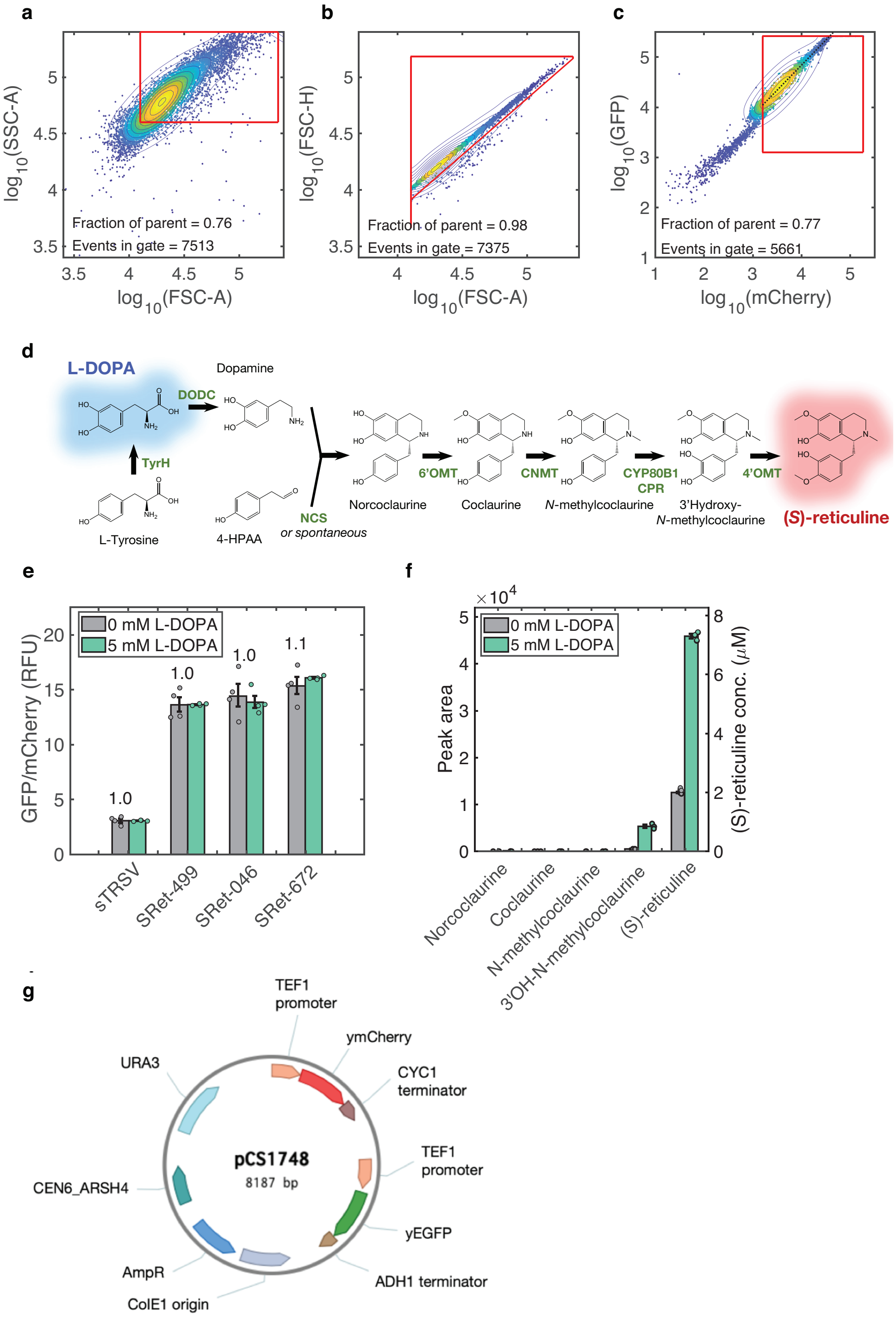

Supplementary Figure 11 | Validation of the *in vivo* gene-regulatory activity of DRIVER-selected biosensors. a. Representative flow cytometry plot of forward scatter area against side scatter area for an aciclovir switch (Acic-493) assayed for gene-regulatory activity in yeast. Each flow cytometry event is shown as a dot. Viable cells are gated with red margins. Viable cells from **a** are shown on the plot of forward scatter height against forward scatter area. **b.** Singlet cells are gated with red margins. **c.** Viable and single cells from **a** and **b** are plotted with GFP against mCherry fluorescence intensities. **d**. Schematic of a heterologous biosynthetic pathway converting L-DOPA, the fed substrate, to (*S*)-reticuline in engineered yeast strain (CSY1171) used to assay the gene-regulatory activities of (*S*)-reticuline biosensors. **e.** Flow cytometry results of (*S*)-reticuline switches assayed in the yeast strain W303α, which does not express the (S)-reticuline biosynthetic pathway, in 0 mM and 5 mM of L-DOPA. sTRSV, wild-type constitutively active ribozyme. Error bars are standard error of mean of n=4 biologically independent samples. **f.** Bar plot of the abundance of BIA pathway intermediates produced in the media from the yeast strain CSY1171 with and without L-DOPA feeding. Peak area refers to the integrated area of the peak detected for each compound by LC-MS/MS using the MRM transitions reported in Materials and Methods. Error bars indicate s.e.m of n=3 biologically independent samples; individual filled circles correspond to biological replicates. Absolute concentration quantities of (S)-reticuline only was obtained from fitting to a standard curve and are indicated on the right vertical axis. **g**. Plasmid map of pCS1748, a dual color fluorescent reporter plasmid used in characterizing ribozyme switch-based biosensors in yeast cells. Biosensor sequences are inserted in the 3' untranslated region via cloning, after the stop codon of yEGFP and before the start of the ADH1 terminator.
