# Supplementary material for: A multiplexed, automated evolution pipeline enables scalable discovery and characterization of biosensors
Source: Nat Commun. 2021 Mar 4;12:1437. doi: 10.1038/s41467-021-21716-0 (PMC7933316; doi:10.1038/s41467-021-21716-0)
Supplement: Supplementary file 6 — Reporting Summary [file 41467_2021_21716_MOESM6_ESM.pdf]

nature research

Corresponding author(s): Christina D. Smolke

Last updated by author(s): Jan 24, 2021

Reporting Summary

Nature Research wishes to improve the reproducibility of the work that we publish. This form provides structure for consistency and transparency in reporting. For further information on Nature Research policies, see our Editorial Policies and the Editorial Policy Checklist.

Statistics

For all statistical analyses, confirm that the following items are present in the figure legend, table legend, main text, or Methods section.

n/a	Confirmed	
☐	☒	The exact sample size (n) for each experimental group/condition, given as a discrete number and unit of measurement
☐	☒	A statement on whether measurements were taken from distinct samples or whether the same sample was measured repeatedly
☐	☒	The statistical test(s) used AND whether they are one- or two-sided<br>*Only common tests should be described solely by name; describe more complex techniques in the Methods section.*
☒	☐	A description of all covariates tested
☐	☒	A description of any assumptions or corrections, such as tests of normality and adjustment for multiple comparisons
☐	☒	A full description of the statistical parameters including central tendency (e.g. means) or other basic estimates (e.g. regression coefficient) AND variation (e.g. standard deviation) or associated estimates of uncertainty (e.g. confidence intervals)
☐	☒	For null hypothesis testing, the test statistic (e.g. F, t, r) with confidence intervals, effect sizes, degrees of freedom and P value noted<br>*Give P values as exact values whenever suitable.*
☒	☐	For Bayesian analysis, information on the choice of priors and Markov chain Monte Carlo settings
☒	☐	For hierarchical and complex designs, identification of the appropriate level for tests and full reporting of outcomes
☒	☐	Estimates of effect sizes (e.g. Cohen's d, Pearson's r), indicating how they were calculated

Our web collection on statistics for biologists contains articles on many of the points above.

Software and code

Policy information about availability of computer code

Data collection	Flow cytometry: MACSQuantify Software version 2.8; Next-generation sequencing: Illumina MiSeq Control Software v3.0, NextSeq Control Software v2.1.0. Surface Plasmon Resonance: Biacore X100 Control software (ver 2.0.2). LC-MS/MS: Agilent MassHunter Workstation LC/MS Data Acquisition for 6400 Series Triple Quadrupole software (ver. B.08.02). Python (ver 3.7). MySQL (ver 5.6)
Data analysis	Custom software used for analyses is available at github.com/btownshend and github.com/jsxiang (see Code availability)<br>Surface Plasmon Resonance: Biacore X100 Evaluation Software (ver 2.0.2).<br>LC-MS/MS: Agilent MassHunter Workstation Qualitative Analysis Navigator software (ver. B.08.00).<br>Gel analysis: ImageJ (ver 2.0.0-rc-68/1.52e)

For manuscripts utilizing custom algorithms or software that are central to the research but not yet described in published literature, software must be made available to editors and reviewers. We strongly encourage code deposition in a community repository (e.g. GitHub). See the Nature Research guidelines for submitting code & software for further information.

Data

Policy information about availability of data

All manuscripts must include a data availability statement. This statement should provide the following information, where applicable:

- Accession codes, unique identifiers, or web links for publicly available datasets
- A list of figures that have associated raw data
- A description of any restrictions on data availability

Biosensor sequences are available through GenBank (accession numbers appear in SI Dataset 3). Aptamer and other short sequences are provided in the Supplementary Information. Source data for all figures can be found in the online version ofare provided with thise paper. All other data are available from the authors on request.

Field-specific reporting

Please select the one below that is the best fit for your research. If you are not sure, read the appropriate sections before making your selection.

- [x] Life sciences
- [ ] Behavioural & social sciences
- [ ] Ecological, evolutionary & environmental sciences

For a reference copy of the document with all sections, see nature.com/documents/nr-reporting-summary-flat.pdf

Life sciences study design

All studies must disclose on these points even when the disclosure is negative.

Sample size	For CleaveSeq experiments, 2 to 3 replicate experiments were used. Considering the high cost of sequencing, more replicates are not used. For qPCR experiments, three technical replicates from separate qPCR wells are used. For flow cytometry experiments, four or more replicates for yeast (saccharomyces cerevisiae) were used, to ensure every experiment has at least 3 successful replicates. Three replicates were used because it is standard practice. For Surface Plasmon Resonance, three or more replicates were used. Due to the large number of individual samples tested in multiple ligand conditions in individual biosensor and ribozyme switch characterization, more replicates were not included. No statistical method was used in sample size calculation.
Data exclusions	No relevant data was excluded.
Replication	Replicates of CleaveSeq experiments were independently transcribed, reverse transcribed and barcoded samples. Replicates are performed in parallel at the same time and each experiment was performed once. Replicates of flow cytometry experiments were carried out on different transformed yeast colonies transformed with the switch containing plasmids, and grown in separate well cultures. All replicates were performed in parallel, and experiments were performed once. Replicates of Surface Plasmon Resonance assays were technical replicates of the same transcribed RNA used in the assay. Each replicate was performed once per day on consecutive, different days, for 3 or more replicates in total. Replicates of LCMS experiments were from different individual yeast colonies transformed with the switch containing plasmids, and grown in separate well cultures. All replicates were performed in parallel, and the experiment was performed once. All attempts at replication were successful.
Randomization	Not relevant because all measurements are comparing biosensor response at varying concentrations of ligand and require self normalization for determining dose response and fold change.
Blinding	Not relevant; negative and positive controls were used as appropriate and the order of the samples performed would not bias the results. Blinding is effectively in place for pooled sequencing experiments.

Reporting for specific materials, systems and methods

We require information from authors about some types of materials, experimental systems and methods used in many studies. Here, indicate whether each material, system or method listed is relevant to your study. If you are not sure if a list item applies to your research, read the appropriate section before selecting a response.

Materials & experimental systems

n/a	Involved in the study	
x		Antibodies
	x	Eukaryotic cell lines
x		Palaeontology and archaeology
x		Animals and other organisms
x		Human research participants
x		Clinical data
x		Dual use research of concern

Methods

n/a	Involved in the study	
x		ChIP-seq
	x	Flow cytometry
x		MRI-based neuroimaging

Eukaryotic cell lines

Policy information about cell lines

Cell line source(s)	Wild-type Saccharomyces cerevisiae strains CEN.PK2-1D and W303 were obtained from EuroSCARF (30000B; 20000A respectively)
Authentication	Cell lines were not independently authenticated by the authors.
Mycoplasma contamination	Yeast cell lines were not tested for mycoplasma contamination.
Commonly misidentified lines (See ICLAC register)	No commonly misidentified cell lines were used in this study.

Flow Cytometry

Plots

Confirm that:

- [x] The axis labels state the marker and fluorochrome used (e.g. CD4-FITC).
- [x] The axis scales are clearly visible. Include numbers along axes only for bottom left plot of group (a 'group' is an analysis of identical markers).
- [x] All plots are contour plots with outliers or pseudocolor plots.
- [x] A numerical value for number of cells or percentage (with statistics) is provided.

Methodology

Sample preparation	For yeast cells, cells in YNB-Ura were diluted 15X into 1XPBS+1% BSA, before being filtered through a 40 µm nylon mesh and run on the flow cytometer.
Instrument	MACSQuant VYB (Miltenyi Biotec, Bergisch Gladbach, Germany)
Software	Custom code was used to analyze flow cytometry data and can be found at https://github.com/jsxiang/FlowAnalysis.
Cell population abundance	Data for at least 2000 yeast cells were collected.
Gating strategy	Analysis of flow cytometry data was performed using a custom MATLAB script, available at https://github.com/jsxiang/FlowAnalysis. For analysis of yeast cells, cells were gated for viable (4.1<log10(FSC-A)<5.4, and 4.6< log10(SSC-A)<5.4) and singlets (log10(FSC-H)<0.65*log10(FSC-A)), and transformed cells (mCherry > 10^3.2 and GFP > 10^3.1 fluorescence units) were used in downstream analysis.

- [x] Tick this box to confirm that a figure exemplifying the gating strategy is provided in the Supplementary Information.
