# Supplementary material for: A multiplexed, automated evolution pipeline enables scalable discovery and characterization of biosensors
Source: Nat Commun. 2021 Mar 4;12:1437. doi: 10.1038/s41467-021-21716-0 (PMC7933316; doi:10.1038/s41467-021-21716-0)
Supplement: Supplementary file 7 — Description of Additional Supplementary Files [file 41467_2021_21716_MOESM7_ESM.pdf]

Title: Supplementary Data 1
Description: Ligands used during selection. Each ligand was used in mixtures that were modified as selection progressed to reduce or remove components for which sensors had been identified. Selections were run in four sets: S1 (with one mixture), S2 (with three mixtures), S3 (initially with four mixtures, combined to three mixtures as selection progressed), and S4 (with one mixture).

Title: Supplementary Data 2
Description: SPR Data. Binding affinity measurements of DRIVER-selected biosensors using surface plasmon resonance.

Title: Supplementary Data 3
Description: In vivo data. Flow cytometry measurements of biosensors as gene-regulatory switches of relative fluorescent reporter expression in yeast cells. Two-tailed, unpaired t-tests were used in determining statistical significance of switching fold activation.

Title: Supplementary Data 4
Description: NGS-identified sensors. Sequence and summary data for each of the biosensors identified.
